# Associations Between Social Risk Factors and Surgical Site Infections After Colectomy and Abdominal Hysterectomy

**DOI:** 10.1001/jamanetworkopen.2019.12339

**Published:** 2019-10-02

**Authors:** Andrew C. Qi, Kate Peacock, Alina A. Luke, Abigail Barker, Margaret A. Olsen, Karen E. Joynt Maddox

**Affiliations:** 1 Washington University School of Medicine in St Louis, St Louis, Missouri; 2 Brown School of Social Work, Washington University in St Louis, St Louis, Missouri

## Abstract

**Importance:**

Surgical site infection (SSI) is an important patient safety outcome. Although social risk factors have been linked to many adverse health outcomes, it is unknown whether such factors are associated with higher rates of SSI.

**Objectives:**

To determine whether social risk factors, including race/ethnicity, insurance status, and neighborhood income, are associated with higher rates of SSI after colectomy or abdominal hysterectomy, 2 surgical procedures for which SSI rates are publicly reported and included in pay-for-performance programs by Medicare and other groups.

**Design, Setting, and Participants:**

This cross-sectional study analyzed adults undergoing colectomy or abdominal hysterectomy, as captured in State Inpatient Databases for Arizona, Florida, Iowa, Massachusetts, Maryland, New York, and Vermont. Operations were performed in 2013 through 2014 at general acute care hospitals in the United States. Data analysis was conducted from October 2018 through June 2019.

**Exposures:**

Colectomy or hysterectomy.

**Main Outcomes and Measures:**

Postoperative complex SSI rates.

**Results:**

A total of 149 741 patients met the inclusion criteria, including 90 210 patients undergoing colectomies (mean [SD] age, 63.4 [15.6] years; 49 029 [54%] female; 74% white, 11% black, 9% Hispanic, and 5% other or unknown race/ethnicity) and 59 531 patients undergoing abdominal hysterectomies (mean [SD] age, 49.8 [11.8] years; 100% female; 52% white, 26% black, 14% Hispanic, and 8% other or unknown race/ethnicity). In the colectomy cohort, 34% had private insurance, 52% had Medicare, 9% had Medicaid, and 5% had other or unknown insurance or were uninsured; 24% were from the lowest quartile of median zip code income. In the hysterectomy cohort, 57% had private insurance, 16% had Medicare, 19% had Medicaid, and 3% had other or unknown insurance or were uninsured; 27% were from the lowest-income zip codes. Within 30 days of surgery, SSI rates were 2.55% for the colectomy cohort and 0.61% for the hysterectomy cohort. For colectomy, black race (adjusted odds ratio [AOR], 0.71; 95% CI, 0.61-0.82) was associated with lower odds of SSI, whereas Medicare (AOR, 1.25; 95% CI, 1.10-1.41), Medicaid (AOR, 1.23; 95% CI, 1.06-1.44), and low neighborhood income (AOR, 1.14; 95% CI, 1.01-1.29) were associated with higher odds of SSI. For hysterectomy, no social risk factors that were examined in this study had statistically significant associations with SSI after adjustment for clinical risk.

**Conclusions and Relevance:**

Inconsistent associations between social risk factors and SSIs were found. For colectomy, infection prevention programs targeting low-income groups may be important for reducing disparities in this postoperative outcome, and policy makers could consider taking social risk factors into account when evaluating hospital performance.

## Key Points

**Question:**

Are social risk factors, including race/ethnicity, insurance status, and neighborhood income, associated with higher rates of surgical site infection (SSI) after colectomy or abdominal hysterectomy, 2 surgical procedures for which SSI rates are publicly reported and included in pay-for-performance programs nationally?

**Findings:**

In this cross-sectional study of 149 741 participants, Medicaid insurance status (a marker for poverty) and living in a low-income zip code were associated with higher SSI rates after colectomy, even after adjusting for clinical risk. For hysterectomy, no social risk factors that were examined in this study had statistically significant associations with SSI after adjustment for clinical risk.

**Meaning:**

For colectomy, infection prevention programs targeting low-income groups may be important for reducing disparities, and policy makers could consider taking social risk into account when evaluating hospital performance.

## Teaser

This cross-sectional study examines whether social risk factors, including race/ethnicity, insurance status, and neighborhood income, are associated with higher rates of surgical site infection after colectomy or abdominal hysterectomy.

## Introduction

Surgical site infections (SSIs) are associated with significant morbidity, mortality, and costs after surgical procedures.^1,2^ Complicating up to 5% of surgical procedures nationally, SSIs are common and often preventable. Consequently, reducing SSIs nationally is a priority for patient safety efforts led by The Joint Commission, the Centers for Medicare & Medicaid Services, and consumer organizations such as the Leapfrog Group.

One strategy these organizations have used to encourage reductions in SSI is public reporting of hospital performance. For example, Hospital Compare, Medicare’s public reporting website, and the Leapfrog Group publish hospitals’ SSI rates for consumers to view. Another strategy to reduce SSI incidence is through the use of financial incentives. For example, Medicare’s Hospital-Acquired Conditions Reduction Program (HACRP), created under the 2010 Patient Protection and Affordable Care Act, is a pay-for-performance program focused on infections and other adverse safety events.^3^ Under this program, 85% of hospitals’ performance scores are determined by infection metrics from the Centers for Disease Control and Prevention’s National Healthcare Safety Network (NHSN), including complex SSI after colectomy and abdominal hysterectomy. Under the HACRP, hospitals in the worst-performing quartile are penalized 1% of their total Medicare payments, amounting to more than 1 billion dollars in its first 4 years.^3^

Consequently, the risk-adjustment methods used for SSI have significant consequences, both in terms of public reputation and financial stability for hospitals. Prior work^4,5^ suggests that teaching hospitals and safety-net hospitals are disproportionately penalized under the HACRP, leading to concerns that performance scores may be driven by patient characteristics outside hospitals’ control, rather than the quality of care that hospitals deliver. Although the NHSN has recently incorporated diabetes, sex, age, and obesity into their SSI risk adjustment models, no adjustment is made for social risk factors, despite prior studies^6,7,8^ showing that social risk factors are associated with an increased risk of infection for other surgical procedures.

Understanding whether social risk factors are associated with SSI for colectomy and abdominal hysterectomy, and whether accounting for these factors would meaningfully change hospitals’ performance on these measures, has implications for public reporting and value-based payment models, but has not previously been described, to our knowledge. Therefore, in this study, we aimed to determine whether SSI rates after colectomy or abdominal hysterectomy differ by patient race/ethnicity, neighborhood income, or insurance type. We also modeled the potential outcomes of accounting for social risk factors on relative performance for safety-net hospitals and teaching hospitals compared with their peers.

## Methods

### Data

Patients undergoing colectomy or abdominal hysterectomy during the years 2013 and 2014 were identified using data from the State Inpatient Databases^9^ for Arizona, Florida, Iowa, Massachusetts, Maryland, New York, and Vermont. These states were selected because they allow linkage of individual patients across time, allowing for the ascertainment of postoperative events that happen either during the index hospitalization or after discharge. We identified procedures using *International Classification of Diseases*, *Ninth Revision*, *Clinical Modification* (*ICD-9-CM*) procedure codes from inpatient stays at general acute care hospitals, in particular the *ICD-9-CM* procedure codes defined by the NHSN for colon surgery and abdominal hysterectomy. We included all individuals aged 18 to 109 years.

Patients were excluded for any of the following reasons: residence in a state other than that where the surgery was performed; additional procedure at a higher priority level performed during the eligible surgery admission, as defined by the NHSN risk of SSI^10^; SSI present at admission (ie, *ICD-9-CM *codes 998.51 or 998.59 coded in the first listed diagnosis or coded as present at admission); or missing information on hospital characteristics from the American Hospital Association Annual Survey.^11^ We restricted analyses to the first surgical procedure per patient, per procedure type.

This study was approved by the Washington University School of Medicine Human Research Protection Office. The requirement for informed consent was waived because of the deidentified, retrospective nature of the data. This study follows the Strengthening the Reporting of Observational Studies in Epidemiology (STROBE) reporting guideline.

### Social Risk Factors

Our primary risk factors were 3 sets of social risk variables: race/ethnicity (white, black, Hispanic, and other or unknown), insurance status (Medicare, Medicaid, private, and other, unknown, or uninsured), and median income for patient zip code in quartiles, all as defined in the Healthcare Cost and Utilization Project data.^9^ Individuals missing data for key risk factors were excluded. Additional variables used for risk adjustment included age, sex (colon only), obesity, diabetes, and whether the hospital was designated as an oncology specialty hospital, as established by the NHSN for risk adjustment of complex 30-day SSIs for colon and abdominal hysterectomy procedures.^12^ Obesity and diabetes were defined as per the Elixhauser Comorbidity Index.^13^ We also adjusted for overall severity of illness by estimating each patient’s risk of an American Society of Anesthesiologists (ASA) score of 3 or greater, on the basis of the probability derived from a logistic regression model developed in a population undergoing colectomy (M. Saeed, A. Vannucci, and M.A.O., unpublished data, 2019). Oncology specialty hospitals were identified using the American Hospital Association Annual Survey.^11^

### Outcomes

Our primary outcome was the occurrence of a complex SSI. We defined complex SSI as an infection coded during an inpatient hospitalization (index hospitalization or readmission) or requiring operative treatment in an ambulatory surgery facility within 30 days of the index procedure. All other SSIs were categorized as noncomplex and were not outcomes for the purpose of this study.

Complex SSIs were identified using *ICD-9-CM* diagnosis and procedure codes within 30 days postoperatively, as described elsewhere.^14,15^ Briefly, SSIs recorded during the first 30 days after surgical procedures were identified using *ICD-9-CM* diagnosis codes from encounters in the State Inpatient Databases and State Ambulatory Surgery Databases.^16^ Prior work^17,18^ in this area has suggested that use of these codes has sensitivity similar to that of routine clinical surveillance for detecting complex SSI, but a lower positive predictive value. We censored the observation period to avoid misclassification of SSIs after a subsequent surgery using the NHSN procedure list, as described elsewhere.^15^

### Statistical Analysis

Patient characteristics were described using standard statistical tests (χ^2^ test and independent-samples *t *test) as appropriate, and unadjusted complex SSI rates were calculated. We then ran generalized linear models including NHSN risk adjustment elements (estimated ASA risk score of ≥3, age in 10-year increments, sex, diabetes, obesity, and oncology hospital),^12^ accounting for clustering by hospital, to examine associations between key risk factors and complex SSIs. We did this in a stepwise manner, such that models first only included each social risk factor alone and subsequently included the NHSN risk adjustment elements and the remainder of the social risk factors.

To determine whether adjusting for social risk factors would affect the relative performance of safety-net or teaching hospitals on these measures of complex SSI, we ranked all hospitals performing at least 20 colectomies on their complex SSI rates and assigned percentiles to each. We defined safety-net hospitals as the quintile of hospitals with the greatest proportion of patients with Medicaid health insurance and teaching hospitals as those reporting a medical school affiliation in the American Hospital Association Annual Survey.^11^ We ranked all hospitals in order of performance on each complex SSI type and calculated the mean percentile score for safety-net and non–safety-net hospitals, as well as teaching and nonteaching hospitals, using no risk adjustment (raw rates), using current NHSN risk adjustment, and after adding social risk factors to the models. Because higher infection rates are worse, a hospital with a percentile score of 0 would be the best performer and a hospital with a percentile score of 100 would be the worst performer. We also examined the proportion that would be in the worst (highest) quartile of performance under each risk adjustment scenario, because the current HACRP program assigns penalties to hospitals in the worst quartile of performance on a broader set of infection measures.

All analyses were performed in SAS Enterprise Guide statistical software version 7.15 HF8 (SAS Institute) or Stata/SE statistical software version 15.1 (StataCorp). Two-sided *P* < .05 was considered statistically significant. Data analysis was conducted October 2018 through June 2019.

## Results

### Patient Population

In total, 149 741 patients met the inclusion criteria, including 90 210 patients undergoing colectomies and 59 531 patients undergoing abdominal hysterectomies (Table 1). The colectomy cohort had a mean (SD) age of 63.4 (15.6) years, and 49 029 (54%) were female; 58% had an estimated ASA score of 3 or higher. Thirteen percent were coded for obesity. White patients composed 74% of the sample, 11% were black, 9% were Hispanic, and 5% were other or unknown race/ethnicity. Medicare was the primary insurer of the cohort (52%), 34% had private insurance, 9% had Medicaid, and 5% had other or unknown insurance or were uninsured; 24% were from the lowest quartile of median zip code income. Within 30 days of surgery, the complex SSI rate was 2.55%.

**Table 1.** Patient Characteristics

Characteristic	Patients, No. (%)	
	Colectomy (n = 90 210)	Hysterectomy (n = 59 531)
Age, mean (SD), y	63.4 (15.6)	49.8 (11.8)
Female	49 029 (54)	59 531 (100)
Estimated American Society of Anesthesiologists score of ≥3^a^	52 577 (58)	8486 (14)
Obesity^b^	12 136 (13)	10 481 (18)
Diabetes^b^	17 311 (19)	6502 (11)
Oncology hospital	1319 (1)	1135 (2)
Race/ethnicity		
White	66 324 (74)	30 562 (52)
Black	10 022 (11)	15 001 (26)
Hispanic	8351 (9)	8288 (14)
Other or unknown	4442 (5)	4918 (8)
Insurance		
Private	30 289 (34)	34 183 (57)
Medicare	46 906 (52)	9612 (16)
Medicaid	8131 (9)	11 228 (19)
Other, uninsured, or unknown	4857 (5)	1720 (3)
Median income for zip code		
Highest quartile	23 017 (26)	14 802 (26)
Second quartile	20 599 (23)	12 762 (22)
Third quartile	23 485 (27)	14 508 (25)
Lowest quartile	21 314 (24)	15 866 (27)
Safety-net hospital^c^	15 174 (17)	9475 (16)

^a^ To estimate American Society of Anesthesiologists score, logistic regression was used to identify people more likely to have a score of 3 or higher.

^b^ Elixhauser Comorbidity Index definitions were used for comorbidities.

^c^ Safety-net hospitals were defined as the quintile of hospitals with the highest percentage of Medicaid patients.

The hysterectomy cohort was younger, with a mean (SD) age of 49.8 (11.8) years, and all were female. Fourteen percent had an estimated ASA score of 3 or higher, and 18% were coded for obesity. Fifty-two percent of the sample were white, 26% were black, 14% were Hispanic, and 8% were other or unknown race/ethnicity. The predominant insurer was private insurance (57% of patients), 16% had Medicare, 19% had Medicaid, and 3% had other or unknown insurance or were uninsured; 27% were from the lowest-income zip codes. Within 30 days of surgery, the complex SSI rate was 0.61%.

### Association Between Social Risk Factors and Rates and Odds of Complex SSI

For the colectomy cohort, in unadjusted analyses, black race was associated with lower raw rates and lower odds of complex SSI (rate for black patients, 2.27%; rate for white patients, 2.62%; unadjusted odds ratio [OR], 0.86; 95% CI, 0.75-0.99). Compared with patients with private insurance, patients with Medicaid insurance had higher odds of complex SSI (OR, 1.32; 95% CI, 1.14-1.52), as did patients from areas with low neighborhood income compared with patients from areas with high neighborhood income (OR 1.20; 95% CI, 1.06-1.35) (Table 2). These associations persisted after risk adjustment (adjusted OR [AOR] for black race, 0.71 [95% CI, 0.61-0.82]; AOR for Medicaid, 1.23 [95% CI, 1.06-1.44]; AOR for low neighborhood income, 1.14 [95% CI, 1.01-1.29]) (Figure). Medicare insurance was not associated with a higher odds of SSI in the unadjusted analysis (unadjusted OR, 0.98; 95% CI, 0.90-1.08) (Table 2) but was associated with higher odds of SSI after risk adjustment (AOR, 1.25; 95% CI, 1.10-1.41) (Figure).

**Table 2.** Unadjusted Rates and Odds of SSI

Characteristic	Colectomy		Hysterectomy	
	Unadjusted SSI Rate, %^a^	Unadjusted OR (95% CI)	Unadjusted SSI Rate, %^a^	Unadjusted OR (95% CI)
Patients, No.	2.55		0.61	
Age, mean, y		0.99 (0.99-0.99)		1.01 (1.00-1.02)
Female	2.50	0.97 (0.89-1.05)	0.61	
Estimated American Society of Anesthesiologists score^b^				
1-2	2.37	1 [Reference]	0.50	1 [Reference]
≥3	2.67	1.13 (1.04-1.23)	1.31	2.64 (2.11-3.30)
Obesity^c^				
No	2.42	1 [Reference]	0.51	1 [Reference]
Yes	3.33	1.39 (1.24-1.55)	1.11	2.19 (1.75-2.73)
Diabetes^c^	2.94	1.21 (1.09-1.33)	1.32	2.53 (1.98-3.22)
Oncology hospital				
Yes	3.11	1.23 (0.90-1.69)	1.23	2.06 (1.21-3.53)
No	2.54	1 [Reference]	0.60	1 [Reference]
Race/ethnicity				
White	2.62	1 [Reference]	0.58	1 [Reference]
Black	2.27	0.86 (0.75-0.99)	0.78	1.34 (1.06-1.70)
Hispanic	2.57	0.98 (0.85-1.14)	0.52	0.89 (0.64-1.24)
Other or unknown	2.00	0.76 (0.61-0.94)	0.49	0.84 (0.55-1.28)
Insurance				
Private	2.47	1 [Reference]	0.51	1 [Reference]
Medicare	2.43	0.98 (0.90-1.08)	0.91	1.78 (1.37-2.30)
Medicaid	3.23	1.32 (1.14-1.52)	0.68	1.32 (1.01-1.74)
Other or uninsured	2.86	1.16 (0.97-1.40)	0.63	1.22 (0.82-1.83)
Median income for zip code				
Highest quartile	2.34	1 [Reference]	0.59	1 [Reference]
Second quartile	2.54	1.09 (0.96-1.23)	0.52	0.88 (0.64-1.21)
Third quartile	2.51	1.08 (0.95-1.21)	0.60	1.02 (0.75-1.39)
Lowest quartile	2.79	1.20 (1.06-1.35)	0.75	1.27 (0.96-1.70)

Abbreviations: OR, odds ratio; SSI, surgical site infection.

^a^ Displayed SSI rates are raw (unadjusted) for each group.

^b^ To estimate American Society of Anesthesiologists score, logistic regression was used to identify people more likely to have a score of 3 or higher.

^c^ Elixhauser Comorbidity Index definitions were used for comorbidities.

**Figure.**
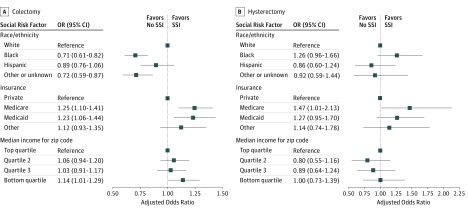
Adjusted Odds of Surgical Site Infection (SSI) by Social Risk Factor Adjusted odds ratios (ORs) (squares) and 95% CIs (horizontal lines) for SSI after colectomy (A) and hysterectomy (B).

For the hysterectomy cohort, in unadjusted analyses, black race compared with white race (OR, 1.34 [95% CI, 1.06-1.70]) and Medicare or Medicaid coverage compared with private insurance (OR, 1.78 [95% CI, 1.37-2.30] and OR, 1.32 [95% CI, 1.01-1.74], respectively) were associated with higher odds of complex SSI (Table 2). After adjustment, only Medicare coverage (AOR, 1.47 [95% CI, 1.01-2.13]) remained statistically significantly associated with higher odds of SSI (Figure).

### Outcomes of Adjusting for Social Risk Factors on Safety-Net and Teaching Hospitals’ Performance on SSI

For colectomy, safety-net hospitals had similar unadjusted complex SSI rates compared with non–safety-net hospitals (2.31% [95% CI, 1.92%-2.71%] vs 2.52% [95% CI, 2.35%-2.69%]; *P* = .31) (Table 3). The mean (SD) percentile score was 46.1 (31.3) among safety-net hospitals (suggesting their performance as a group was worse than the median) and 50.2 (28.2) for non–safety-net hospitals (suggesting their performance as a group was slightly better than the median), although these were not statistically different from one another (*P* = .21 for comparison). Similarly, 23.9% of safety-net hospitals were in the worst quintile of infection rates compared with 24.2% of non–safety-net hospitals (difference, −0.3%; 95% CI, −9.9% to 9.4%; *P* = .96 for comparison). Adding current NHSN adjustment or adding social risk adjustment did not change these patterns (under full adjustment, mean [SD] percentile, 48.3 [32.9] vs 49.8 [27.9], *P* = .70; proportion in the worst quartile, 28.3% vs 23.0% [difference, 5.3%; 95% CI, −4.8% to 15.3%], *P* = .28).

**Table 3.** Percentile Performance for Safety-Net and Teaching Hospitals^a^

Variable	Unadjusted						NHSN Adjusted				NHSN and Social Risk Factor Adjusted			
	Rate, %	*P* Value	Rank	*P* Value	Worst Quartile, %	*P* Value	Rank	*P* Value	Worst Quartile, %	*P* Value	Rank	*P* Value	Worst Quartile, %	*P* Value
Colectomy														
Overall	2.49		49.5		24.1		49.5		23.9		49.5		23.9	
Safety-net hospital														
Yes	2.31	.31	46.1	.21	23.9	.96	49.1	.89	30.4	.11	48.3	.70	28.3	.28
No	2.52		50.2		24.2		49.6		22.5		49.8		23.0	
Teaching hospital														
Yes	2.39	.28	48.6	.54	20.1	.08	50.0	.72	23.4	.83	49.7	.88	21.5	.29
No	2.55		50.1		26.9		49.1		24.3		49.3		25.6	
Hysterectomy														
Overall	0.56		49.7		24.2		49.7		23.9		49.7		23.9	
Safety-net hospital														
Yes	0.69	.18	52.9	.17	26.8	.49	52.2	.28	25.8	.63	53.5	.10	30.9	.07
No	0.52		48.8		23.4		49.0		23.4		48.6		21.8	
Teaching hospital														
Yes	0.63	.16	55.0	<.001	27.6	.15	55.3	<.001	29.2	.02	55.0	<.001	28.7	.04
No	0.50		45.5		21.5		45.3		19.7		45.5		20.2	

Abbreviation: NHSN, National Healthcare Safety Network.

^a^ Higher ranks are worse, because higher infection rates are worse. Median performance is the 50th percentile. Current NHSN adjustment includes diabetes, estimated American Society of Anesthesiologists risk score, age, sex, obesity, and whether the patient is hospitalized at an oncology hospital.

For colectomy, unadjusted SSI rates at teaching hospitals were similar to those for nonteaching hospitals (2.39% [95% CI, 2.19%-2.59%] vs 2.55% [95% CI, 2.33%-2.78%]; *P* = .28), as were relative percentiles (mean [SD], 48.6 [26.1] vs 50.1 [30.5]; *P* = .54) and proportion in the worst quartile (20.1% vs 26.9% [difference, −6.8%; 95% CI, −14.2% to 0.6%]; *P* = .08) (Table 3). Adding current NHSN adjustment or adding social risk adjustment narrowed the differences (under full adjustment, mean [SD] percentile 49.7 [27.0] vs 49.3 [30.1], *P* = .88; proportion in the highest quartile, 21.5% vs 25.6% [difference, −4.1%; 95% CI, −11.5% to 3.4%], *P* = .29).

Patterns were similar for hysterectomy: safety-net hospitals had similar complex SSI rates compared with non–safety-net hospitals (0.69% [95% CI, 0.46%-0.91%] vs 0.52% [95% CI, 0.42%-0.62%], *P* = .18; mean [SD] performance percentile, 52.9 [26.7] vs 48.8 [25.3], *P* = .17; 26.8% vs 23.4% in the worst quartile [difference, 3.4%; 95% CI, −6.5% to 13.4%], *P* = .49) (Table 3). However, in this case, although adjustment for current NHSN risk factors did not change rates significantly, adjustment for social risk factors widened gaps in performance (mean [SD] performance percentile, 53.5 [27.3] vs 48.6 [25.1], *P* = .10; 30.9% vs 21.8% in the worst quartile [difference, 9.1%; 95% CI, −1.1% to 19.4%], *P* = .07).

Comparing teaching with nonteaching hospitals for hysterectomy, complex SSI rates were similar (0.63% [95% CI, 0.51%-0.75%] vs 0.50% [95% CI, 0.37%-0.64%], *P* = .16) (Table 3), but teaching hospitals had higher (worse) percentile ranks (mean [SD] performance percentile, 55.0 [25.3] vs 45.5 [25.2], *P* < .001) and a similar proportion in the worst quartile (27.6% vs 21.5% [difference, 6.1%; 95% CI, −2.2% to 14.4%], *P* = .15). These patterns became more marked under NHSN or full adjustment (under current NHSN adjustment, proportion in the worst quartile, 29.2% vs 19.7% [difference, 9.5%; 95% CI, 1.1%-17.8%], *P* = .03; under full adjustment, proportion in the worst quartile, 28.6% vs 20.2% [difference, 8.5%; 95% CI, 0.2%-16.8%], *P* = .04).

## Discussion

We found that social risk factors were inconsistently associated with complex SSI rates after colectomy or hysterectomy. For colectomy, Medicaid status (a marker for poverty) and living in a low-income zip code were associated with higher complex SSI rates. For hysterectomy, no social risk factors that we examined in this study had statistically significant associations with SSI. Safety-net hospitals performed similarly to non–safety-net hospitals on measures of complex SSI for both colectomy and hysterectomy, and teaching hospitals performed similarly to nonteaching hospitals. Adding social risk factors to current risk adjustment methods for complex SSI did not change safety-net hospitals’ relative performance under the program, although it widened the gap between teaching and nonteaching hospitals.

Patients with either individual or community markers for poverty had higher complex SSI rates for colectomy. Although prior studies^19,20^ have demonstrated that several clinical factors are associated with SSI rates in this procedure, including more-complex operations and more-complex patients, open vs laparoscopic procedures, diabetes, and obesity, as well as tobacco and alcohol abuse, there are no prior studies, to our knowledge, examining social risk factors in the context of colectomy. Our findings should be considered exploratory, but if they are confirmed, they may suggest that patients from disadvantaged backgrounds could benefit from additional targeted preoperative care and postoperative monitoring to reduce the risk of complex SSI. Interventions such as surgical bundles, optimization of diabetes control, and close postoperative surveillance could help reduce these disparities. In addition, public reporting and pay-for-performance programs that measure hospital performance according to hospital SSI rate could consider evaluating whether the addition of social risk to risk adjustment models could improve their accuracy. Because patient and neighborhood income levels are outside the control of hospitals, such adjustment could yield fairer performance comparisons, while encouraging all hospitals to focus infection prevention efforts on their highest-risk populations.

We did not find consistent associations between social risk and complex SSI for hysterectomy. Similar to colectomy, prior studies^21,22^ have identified complex surgery, open vs laparoscopic approach, diabetes, and obesity as risk factors for SSI for this procedure. Rates of complex SSI after hysterectomy are very low—0.61%, or roughly 1 in 200 operations, in our sample—which may make identification of differences associated with any particular traits more difficult. This may also speak to the difficulty in using such rare events as quality measures, because a single infection could change hospitals’ measured performance from exemplary to poor. The use of multiple types of infections in NHSN and other programs may mitigate this problem to some degree, however.

Some differences in SSI rates by social risk were reduced in magnitude after risk adjustment, suggesting that there were medically mediated associations between social risk and SSI. Although Medicare insurance is not considered a social risk factor (because all US individuals regardless of income are eligible to enroll at age 65 years), it is a marker of age and medical complexity and was consistently associated with higher SSI risk; this raises the possibility of unmeasured confounding, because the NHSN risk adjustment is minimal compared with other clinical outcome measures used in the Medicare program. Further research should examine whether more-robust risk adjustment might explain some of the risk we found to be associated with local or community poverty and whether better risk adjustment could improve these measures’ fairness in measuring hospital performance.

### Limitations

This study has limitations. We relied on billing data for event ascertainment and for risk adjustment and had data for a limited number of states. The NHSN SSI data are drawn from a different set of hospitals nationally, and NHSN surveillance is based on clinical data sourced from primary records and active hospital-level surveillance rather than billing data; therefore, although we are examining the same clinical event, our rates and patterns may not be identical to theirs. Our risk adjustment may also be subject to similar limitations in coding sensitivity for comorbidities as our outcome measure. Event rates, especially for hysterectomy, were very low, which may suggest limited sensitivity of our analysis for SSI events and likely limited our power in terms of identifying significant associations between social risk factors and our outcome. Our use of an inpatient and ambulatory surgical database to identify events may also contribute to the low observed incidence, because although we expect most complex SSI events included in the outcome to be managed in this setting, some may be managed exclusively in the outpatient setting and, thus, would be excluded from our analysis. This could introduce bias if lower-income populations were more likely to be managed in the outpatient setting, although we are aware of no evidence that this is the case. We simulated percentile ranking to illustrate the outcomes of additional risk adjustment, but the HACRP and other patient safety programs also include other safety measures, so our findings do not directly reflect performance or payment under these programs more broadly. Finally, we had data on only a limited set of social determinants of health, and our negative findings particularly for hysterectomy should not be taken to suggest that no social risk factors are associated with postsurgical outcomes; further research could address additional social risk factors such as education or employment, as well as associated ones such as health literacy.

## Conclusions

We found inconsistent associations between social risk and complex SSI. For colectomy, we identified specific patient populations, such as individuals with Medicaid coverage and those in low-income areas, that could be targeted to reduce infection rates. For colectomy, consideration could also be given to adjusting SSI rates for social risk factors in public reporting programs or pay-for-performance programs like the HACRP. The outcomes of patient safety–focused pay-for-performance programs on health care systems serving socially at-risk populations should be closely monitored to identify disparities and opportunities for improvement.
